# Resilience of people with chronic medical conditions during the COVID-19 pandemic: a 1-year longitudinal prospective survey

**DOI:** 10.1186/s12888-022-04265-8

**Published:** 2022-10-01

**Authors:** Lorenzo Tarsitani, Irene Pinucci, Federico Tedeschi, Martina Patanè, Davide Papola, Christina Palantza, Ceren Acarturk, Emma Björkenstam, Richard Bryant, Sebastian Burchert, Camille Davisse-Paturet, Amanda Díaz-García, Rachel Farrel, Daniela C. Fuhr, Brian J. Hall, Anja C. Huizink, Agnes Iok Fong Lam, Gülşah Kurt, Ingmar Leijen, Ellenor Mittendorfer-Rutz, Naser Morina, Catherine Panter-Brick, Fredrick Dermawan Purba, Soledad Quero, Soraya Seedat, Hari Setyowibowo, Judith van der Waerden, Massimo Pasquini, Marit Sijbrandij, Corrado Barbui

**Affiliations:** 1 Department of Human Neurosciences, Sapienza University of Rome, Rome, Italy; 2 Department of Clinical, Neuro-, and Developmental Psychology and WHO Collaborating Center for Research and Dissemination of Psychological Interventions, Vrije Universiteit, Amsterdam, the Netherlands; 3 WHO Collaborating Centre for Research and Training in Mental Health and Service Evaluation, Department of Neuroscience, Biomedicine and Movement Sciences, University of Verona, Verona, Italy; 4 Department of Psychology, Koc University, Istanbul, Turkey; 5 Department of Clinical Neuroscience, Division of Insurance Medicine, Karolinska Institutet, Berzelius väg 3, 17177 Stockholm, Sweden; 6 School of Psychology, University of New South Wales, Sydney, Australia; 7 Department of Education and Psychology, Division of Clinical Psychological Intervention, Freie Universität Berlin, Berlin, Germany; 8 Université Paris-Saclay, UVSQ, Inserm, CESP, 94807 Villejuif, France; 9 Department of Psychology and Sociology, Universidad de Zaragoza (Teruel), Teruel, Spain; 10 Department of Anthropology, Yale University, New Haven, USA; 11 Department of Health Services Research and Policy, London School of Hygiene and Tropical Medicine, Tavistock Place, London, UK; 12 Center for Global Health Equity, NYU Shanghai, Shanghai, People’s Republic of China; 13 New York University School of Global Public Health, New York, NY USA; 14 Department of Clinical, Neuro-, and Developmental Psychology, Vrije Universiteit, Amsterdam, the Netherlands; 15 Centre for Macau Studies, University of Macau, Macau, SAR People’s Republic of China; 16 Department of Communications, University of Macau, Macau, SAR People’s Republic of China; 17 Department of Marketing, School of Business and Economics, Vrije Universiteit, Amsterdam, The Netherlands; 18 Department of Consultation-Liaison Psychiatry and Psychosomatic Medicine, University Hospital of Zurich, University of Zurich, Zurich, Switzerland; 19 Jackson School for Global Affairs, Yale University, New Haven, USA; 20 Faculty of Psychology, Universitas Padjadjaran, Jatinangor, Indonesia; 21 Department of Basic, Clinical Psychology and Psychobiology, Universitat Jaume I, Castellón, Spain; 22 CIBER de Fisiopatología de la Obesidad y Nutrición (CIBEROBN), Carlos III Institute of Health, Madrid, Spain; 23 Department of Psychiatry, Faculty of Medicine and Health Sciences, Stellenbosch University, Cape Town, South Africa; 24 INSERM U1136, Sorbonne Université, Institut Pierre Louis d’Épidémiologie et de Santé Publique, Social Epidemiology Research Team, Paris, France

**Keywords:** Resilience, COVID-19 pandemic, Chronic medical conditions, Stress

## Abstract

**Backgrounds:**

Individuals with chronic medical conditions are considered highly exposed to COVID-19 pandemic stress, but emerging evidence is demonstrating that resilience is common even among them. We aimed at identifying sustained resilient outcomes and their predictors in chronically ill people during the first year of the pandemic.

**Methods:**

This international 4-wave 1-year longitudinal online survey included items on socio-demographic characteristics, economic and living situation, lifestyle and habits, pandemic-related issues, and history of mental disorders. Adherence to and approval of imposed restrictions, trust in governments and in scientific community during the pandemic were also investigated. The following tools were administered: the Patient Health Questionnaire, the Generalized Anxiety Disorder scale, the PTSD Checklist DSM-5, the Oslo Social Support Scale, the Padua Inventory, and the Portrait Values Questionnaire.

**Results:**

One thousand fifty-two individuals reporting a chronic condition out of 8011 total participants from 13 countries were included in the study, and 965 had data available for the final model. The estimated probability of being “sustained-resilient” was 34%. Older male individuals, participants employed before and during the pandemic or with perceived social support were more likely to belong to the sustained-resilience group. Loneliness, a previous mental disorder, high hedonism, fear of COVID-19 contamination, concern for the health of loved ones, and non-approving pandemic restrictions were predictors of not-resilient outcomes in our sample.

**Conclusions:**

We found similarities and differences from established predictors of resilience and identified some new ones specific to pandemics. Further investigation is warranted and could inform the design of resilience-building interventions in people with chronic diseases.

**Supplementary Information:**

The online version contains supplementary material available at 10.1186/s12888-022-04265-8.

## Introduction

The Coronavirus Disease 2019 (COVID-19) pandemic led to unprecedented community-wide mandatory lockdowns and isolation measures worldwide. Enduring restrictions and lockdowns, stay-at-home orders, social isolation, financial loss, physical distancing and fear of contamination were immediately acknowledged as unpredictable and uncontrollable parts of a unique and widespread stressor, with clear personal salience [19, 62]. Many studies have documented elevated stress reactions to the COVID-19 pandemic [74, 98]. Recent systematic reviews manly based on cross-sectional and uncontrolled studies reported a detrimental effect of the pandemic on mental health with clinically significant stress, anxiety, and depressive symptoms in approximately one third of non-clinical samples [50, 73]. Another review of studies of the general public revealed higher level of symptoms of anxiety and depression severity compared to the period to before COVID-19 [92]. However, a meta-analysis of 25 longitudinal studies and natural experiments on the impact of COVID-19 lockdowns on mental health, showed that the effect is small and highly heterogeneous [66, 71].

In the first year of the COVID-19 pandemic, some authors argued that the effects on mental health would show substantial variation across individuals and that long-term resilience would be the most common outcome worldwide [55, 65]. This concept is in line with a relatively recent shift in research from stress effects to resilience and its determinants [12]. Based on this research, most people are expected to experience a pattern of adaptation to the COVID-19 pandemic and this should be reflected in non-elevated stress levels over time. Resilience refers to the ability of an individual or a group to adapt and maintain mental health in stressful situations or events [1, 32, 39]. Resilient adaptation is prevalent after trauma exposure [26], traumatic disasters and even during a pandemic [13, 65]. A variety of individual characteristics are associated with stress resilience, such as age, gender, copying styles, daily habits and others [36, 70]. Available studies have focused more on potential traumatic events rather than chronic stressors and report a variety of static and dynamic, individual, and socio-environmental factors associated with resilient trajectories. Among them, an interesting area of research studies personal values, defined as guiding principles of individuals in daily life [78]. Values can moderate the relation between stressors and resilience [3]. However, to date, identified variables, according to the so called “Resilience paradox”, are modestly associated and do not adequately predict resilient outcomes [6, 8, 9].

There are two main approaches to the assessment of resilience: the first concerns the use of resilience questionnaires, which usually focus on self-reported individual characteristics as for example personality traits, attitudes, behaviors, and coping strategies. Nevertheless, these tools include restricted sets of variables that usually show poor predictive utility [6, 39]. The second approach is based on analyses of outcome trajectories during exposure to adversity: resilient individuals are those who maintain low or no stress symptoms (referring to e.g. depressive, anxiety, and post-traumatic symptoms) over time and predictors are explored using multivariate analyses [6, 9, 26, 30].

Among vulnerable groups, individuals with chronic medical conditions are considered highly exposed to COVID-19 pandemic stress and resilient trajectories are not expected as the rule in them [97]. Medical illness increases stress-related vulnerability [33, 82, 91]. During lockdowns, other factors, including difficulties and problems in medication access, attending routine visits, and procedures were reported in patients with physical illness [18]. The drastic change in daily life with a reduction of physical activity and other healthy lifestyles may have led to worsening of clinical symptoms and psychological conditions among those with a chronic illness [64]. Additionally, awareness of the higher risk of severe or lethal COVID-19 infection may have produced high levels of fear-related stress in individuals with chronic medical conditions [88]. Indeed, individuals with chronic medical conditions reported increased worry about getting infected with COVID-19 infection [34] and have a higher vulnerability to COVID-19 pandemic-related stress. A poor self-rated health status or a history of chronic illness were significantly associated with a greater psychological impact and higher stress, anxiety and depression scores in the COVID-19 outbreak in China [94, 95]. An online survey conducted during the first phase of the COVID-19 pandemic in Italy reported an association between history of medical problems and increased anxiety and depression [58].

In face of this, emerging evidence is demonstrating that resilient outcomes are frequent even with people who have chronic medical conditions during the pandemic [20]. Individuals with a chronic condition reported more baseline loneliness compared to healthy subjects but did not have a significant increase in loneliness during COVID-19 social distancing measures in a 3-month longitudinal study [49].

Habits of daily life are crucial to achieve resilient outcomes during adversity [35]. COVID-19 isolation measures offer an unprecedented opportunity to study resilience in people deprived of one common buffer against stress: their daily routine. Focusing on people with chronic medical conditions can provide evidence on determinants of resilience in vulnerable individuals. However, to date, no studies have investigated this topic. To address this gap in the literature, this study aimed to empirically identify longitudinal trajectories of resilient outcomes and to explore associated variables at baseline among subjects with chronic medical conditions during the first year of the COVID-19 pandemic. Potential predictors were selected according to the general literature of resilience during adversities [6, 9, 26, 30] and included socio-demographic characteristics, living conditions and habits, changes in working condition, social support and loneliness, and history of mental disorders. Fear of contamination, adherence to- and approval of restrictive measures, trust in government and scientific community were chosen as variables specifically related to COVID-19 pandemic. Among individual characteristics, a measure of personal values was included because they are poorly studied in resilience literature, but they are supposed to interfere with adaptation to pandemic stress [96].

## Methods

### Subjects

This study is part of the COvid MEntal healTh Survey “Mental health effects of the COVID-19 outbreak – a longitudinal international comparison” (COMET) [63], a large international project addressing mental health effects of the COVID-19 pandemic.

This 4-wave longitudinal cross-country online survey included 13 countries affected by the COVID-19 virus outbreak: Australia, France, Germany, Indonesia, Italy, Macau SAR China, The Netherlands, South Africa, Spain, Sweden, Switzerland, Turkey, and United Kingdom. A convenience sample was recruited online via social networks (e.g., Facebook, Instagram, Twitter, WhatsApp etc.), university postings and circular emails. The inclusion criteria were age 18 and older, ability to understand the dominant local language, and an online informed consent agreement to participate in the survey and to be contacted via email for the subsequent waves. Informed consent was obtained from all subjects through a secure web link after the aims of the study and the procedure had been explained at the beginning of the survey. Each participant who agreed to be contacted again received an invitation and personal link to the new versions of the survey. No participants could be added to the list of people invited to the survey after the first wave. In the present study, only adult individuals with at least one chronic medical condition, and contributing to at least three of the four waves, were included. The first wave of the survey took place simultaneously in all countries between May and July 2020. The second wave took place between September and October 2020, the third wave took place in December 2020, while the fourth wave took place between March and April 2021.

### Procedures

The online tool Survalyzer (www.survalyzer.com) was used to administer the survey, with different languages available (Bahasa Indonesia, Cantonese, Dutch, English, French, German, Italian, Spanish, Swedish, Turkish). Participation in the survey was voluntary and participants were free to withdraw from the survey at any time, without giving a reason and without any consequences. A monetary incentive was provided in the form of a lottery (50 euros for ten participants). No identifying information was collected during interviews, and all data were pseudo-anonymized and encrypted. A list of referral options and national contact details for participants who needed any psychosocial support was provided.

The COMET study was approved by the ethical review board of the Faculty of Behavioral and Movement Sciences of the Vrije Universiteit Amsterdam (VCWE-2020-077), by the Ethics Committee of the Department of Human Neurosciences - Sapienza University of Rome, Italy (approval n° 02/2020), the ethical review board of the University of Verona (UNIVR n8/2020), by the ethical review board in Sweden (Dnr 2020–02157), the Research Ethics Committee Universitas Padjadjaran Bandung (431/UN6.KEP/EC/2020) the ethical review board of the Koc University, Turkey (2020.134.IRB3.072), the Health Research Ethics Committee of Stellenbosch University (Ethics Reference No: N20/05/016_COVID-19), the Research Ethics Committee Universitas Padjadjaran Bandung (431/UN6.KEP/EC/2020), and by the ethical review board of Freie Universität Berlin, Germany (023/0000). The French contribution to the COMET consortium is in accordance with French regulations concerning the Comité de Protection des Personnes (CCP), the Règlement Général sur la Protection des Données (RGPD) and the Informatique et Libertés law.

All procedures followed were in accordance with Helsinki Declaration.

### Assessment

The survey included items measuring socio-demographic characteristics, economic and living situation, lifestyle and habits, values, pandemic-related issues, and psychiatric history. Adherence to and approval of imposed restrictions, trust in governments and in scientific community, and changes in working habit during the pandemic were also investigated (see Statistical Analysis section).

Diagnoses of chronic medical conditions were enquired with the following item: “Do you have any of the below mentioned conditions? (You can choose multiple options)”. Choices were: High blood pressure; Heart disease; Pulmonary or Asthmatic diseases; Diabetes; Immune diseases; Cancer; Other (open-ended question). Answers to the question “Other” were screened by two Medical Doctors (IP and LT) and only chronic medical conditions were included (e.g., neurologic, gastrointestinal, nephron-urologic, hematologic, dermatologic, endocrine, gynecological, ophthalmic, and rare genetic disease).

#### Resilience assessment tools

Resilient outcomes were conceptualized according to the previously mentioned approach that is to identify individuals showing no or low stress symptoms over time during adversity [6, 9, 26, 30]. We chose not to use perceived stress scales, which measures anxiety and depressive symptoms that are subjectively attributed to stress by the individuals. We directly assessed the most common manifestations of stress, that are anxiety, depression, and post-traumatic symptoms measuring the effects of COVID-19 pandemic, a shared and unique source of stress at the time of the assessment.

The following tools were administered at all four waves.

##### Patient health questionnaire – 9 items (PHQ-9)

The PHQ-9 is a 9-item self-report questionnaire to screen for depressive symptoms and depressive disorders during the past 2 weeks. Items are on a 0–3 Likert scale with the total score ranging from 0 to 27. Higher scores indicate more depressive symptoms. PHQ-9 shows good psychometric properties with a sensitivity of 0.77 (0.71–0.84) and a specificity of 0.94 (0.90–0.97) [42]. Validated or official versions were available in Chinese [100] English [42], French [17], German [48], Indonesian [90], and Turkish [75]. For Italian, Spanish and Swedish the translated versions downloadable from the PHQ website were used (www.phqscreeners.com).

##### Generalized anxiety disorder scale – 7 items (GAD-7)

The GAD-7 is a 7-item rating scale with each item scoring on a 0–3 scale and a total score ranging from 0 to 21. Higher scores indicate more anxiety symptoms during the past 2 weeks. The GAD-7 scale showed good psychometric properties [80]. Validated or official versions of the GAD-7 were available in English [80], French [59], German [47], Indonesian [14], Spanish [27], and Turkish [41]. For Chinese, Italian and Swedish the translated versions downloadable from the PHQ website were used (www.phqscreeners.com).

##### PTSD checklist DSM-5 – 4 items (PCL-5)

The 4-item PCL- 5 scale was used to measure post-traumatic stress symptoms during the past week according to DSM-5 Post-Traumatic Stress Disorder (PTSD). Items assess symptoms on a 0–4 Likert scale. Higher scores indicate more PTSD symptoms. It is a short version of the PCL-20, one of the most used tools to screen PTSD worldwide, and a valid and reliable measure of PTSD [67]. Validated versions of the PCL-5 were available in English [67], French [2], German [43], Indonesian [84], Swedish [85], and Turkish [11]. The questionnaire was translated to the other used languages through a double-translation and reconciliation process followed by an independent verification of the equivalence between the final versions.

#### Covariates

The following tools were chosen according to the general literature on resilience and considering the likely influence of the fear of contamination during the pandemic. Among individual’s characteristics we addressed personal values to investigate their role in the adaptation to the drastic change of the daily life imposed by the pandemic.

##### Oslo social support scale (OSSS-3)

The Oslo Social Support Scale OSS-3 is a short and valid scale to determine the level of social support. It covers different fields of social support by measuring the number of people the respondent feels close to, the interest and concern shown by others, and the ease of obtaining practical help from others. The OSS-3 scores range from 3 to 14 with a score of 3–8 = poor support; 9–11 = moderate support; and 12–14 = strong support [40]. A validated version of the OSSS-3 was available in English [40]. The questionnaire was translated in the other languages with the same methodology described above. An item investigating the feeling of loneliness (“Do you feel lonely?”) was added to this scale.

##### Padua inventory

We administered the contamination subscale of the Padua Inventory which assesses obsessive compulsive symptoms. The contamination subscale consists of 10 items assessing contamination fear was used in this study. Items are scored on a 5-point Likert-type scale ranging from 0 (not at all) to 4 (very much). The Padua Inventory demonstrated adequate internal consistency [15]. Validated versions of the Padua Inventory were available in English [15], French [38], German [89], Spanish [57], and Turkish [101]. The questionnaire was translated in the other languages with the same methodology as described above.

##### Portrait values questionnaire – 11 items (PVQ-11)

The 11- items PVQ scale is a self-administered questionnaire to measure the same ten basic value orientations measured by the Schwartz Value Survey (power, achievement, hedonism, stimulation, self-direction, universalism, benevolence, tradition, conformity, security). For each portrait, respondents answer: “How much like you is this person?” They check one of six boxes labelled: very much like me, like me, somewhat like me, a little like me, not like me, and not like me at all [78, 99]. The eleven selected items are the ones displayed in the context of the World Values Survey (http://www.worldvaluessurvey.org/wvs.jsp). Validated versions of the PVQ were available in English [78], French [93], German [76], Indonesian [46], and Turkish [23]. The questionnaire was translated in the other languages with the same methodology as described above. Following common procedure when using Schwartz values, scores were ipsatized, i.e. centered on the mean of values for each person in order to assess the relative importance of each value [10, 77].

### Statistical analysis

Clinical and socio-demographic variables were compared between participants included in the present analysis (those completing at least three out of four surveys) and participants who were excluded because they did not complete at least three surveys.

**Fig. 1:**
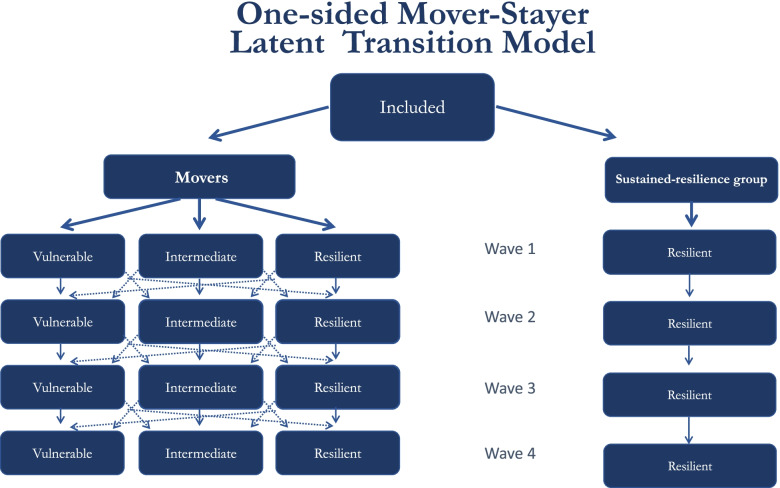
Graphical representation of the Mover-Stayer model

GAD, PHQ and PCL scores were used to develop a resilience indicator. For each of the four time-points, participants could fit into one of three latent classes (“vulnerable”, “intermediate” and “resilient”) based on their values of the GAD, PHQ and PCL scores. Based on this allocation, participants were grouped into those forced to remain into the resilient class in each of the four time-points (stayers, Group 1), and those who were free to move from one class to another in different time-points (movers, Group 2). Specifically, we performed a variant of the Mover-Stayer Latent Transition Analysis model [28] in which stayers (Group 1) were only allowed for the resilient class (for an example of a model where stayers have probability 1 to be in a specific class, see [60]) while movers (Group 2) were allowed to be in any of the three classes at each timepoint. To improve interpretability, indicator means were held equal across time both for each class within Group 2 (“vulnerable”, “intermediate” and “resilient”) and for Group 1 (that was allowed to have a different mean with respect to the subgroup of people in Group 2 turning out to be resilient at each specific time-point), so that class membership had the same meaning at the different timepoints. Based on this approach, four categories of participants were identified: stayers (Group 1), that is participants who showed “sustained-resilience”, and movers (Group 2), that included participants belonging to the “vulnerable” or “intermediate” or “resilient not belonging to the sustained-resilience class” categories at each timepoint. A graphical representation of the model is presented in Fig. 1. Importantly, the Mover-Stayer model restricts only the stayers from moving, while it does not restrict the movers to stay (Fig. 1). Therefore, there is a small probability that some participants, not classified as stayers, actually stayed in the same class across time. This may happen if their observed variable values were on the whole similar to those classified as movers.

Being sustained-resilient was our outcome of interest and was predicted through a logistic regression. In order to take uncertainty of individual class allocation into account, a one-step method procedure was adopted, i.e.: the class solution and the prediction for class membership were estimated simultaneously.

#### Generation of predictor variables

The following sociodemographic predictor variables were included: gender (excluding gender categories not reaching at least 10 individuals, and assigning transgender people to their declared gender; details of gender distribution are given in Additional file 1: Table S5); age in years, education (dichotomized as: “University degree vs at most High School”), working status before and during the pandemic (with two dummy variables for “being employed before and after the pandemic” and “lost job/job stopped during the pandemic”, using the lack of information on employment status as the reference category), square meters of living space per person, and indicator variables for whether COVID-19 had implied an income reduction, presence of a previous mental disorder, not disagreeing with governmental COVID-19 regulations (being at least neutral vs “Disagree” and “Strongly disagree”), whether participants declared to adhere to such regulations “all of the time”, having gone out at least 3 times per week in the previous 2 weeks, knowing anyone who had been infected by COVID-19, whether own job required possible COVID-19 exposure, whether the job of a close person required possible exposure, whether it was difficult getting food, medication or other necessities for self, and whether it was difficult getting food, medication or other necessities for a close person.

We also included, among predictors, a question on whether a person would get vaccinated in case a vaccine was found (“Yes” vs “No”, “Don’t know” and “Refusal to answer”), a latent variable related to conspiracy beliefs about COVID-19 pandemic, built on three binary indicators, i.e. items “Certain significant events have been the results of the activity of a small group who secretly manipulate world events”, “Group of scientists manipulate, fabricate, or suppress evidence in order to deceive the public” and “Governments should let people make their own decisions about how to best protect themselves and their loved ones from the COVID-19 virus”. Such variables were dichotomized, with individuals being at least neutral or replying “Don’t know” or “Refusal to answer” on one side, and those replying “Disagree” and “Strongly disagree” on the other side. Details of the identification strategy of the conspiracy belief latent construct are described in the Additional file 1.

The following clinical predictors were additionally considered: Padua Inventory and OSSS scale scores; OSSS item: “Do you feel lonely?” (dichotomized as “Often” and “Frequently” vs at most “Sometimes”); the centered values of eight PVQ dimensions (to avoid collinearity issues, two were excluded): achievement, hedonism, stimulation, self-direction, benevolence, tradition, conformity and security; dummy variables for whether each chronic condition was present: pulmonary disease/asthma, diabetes, immunological disorders, cancer, heart disease/hypertension, other chronic condition.

For clinical scales, in case of missing items, the Corrected Item Mean Substitution method [37] was used (i.e. the item mean across participants weighted by the subject’s mean of completed items), using information from other individuals included in the analysis. The substitution was only performed for observations having less than 50% of missing items; moreover, estimated values above the maximum or below the minimum of the range of possible values were set to the maximum and minimum, respectively.

All variables were measured at the first wave and all psychometric tools were administered in all the four waves. Willingness to be vaccinated and conspiracy beliefs indicators were included from the second wave.

In all models, in order to avoid listwise deletion of observations with missing values either in the resilience or in the conspiracy indicators, the Full Information Maximum Likelihood approach was adopted. Details of the model estimation strategy are given in the Additional file 1. Analyses were performed using MPlus [61] and Stata 17 [81].

## Results

The number of adult participants was 8011. Among them, 1993 (24.9%) had at least one chronic medical condition, and 1052 (13.1%) contributed to at least three out of four survey waves, and were therefore included in this study. In comparison with excluded participants, participants presenting at least one chronic medical condition showed worse mean values at the first wave in each item of scales used to assess anxiety, depression and post-traumatic stress (Additional file 1: Table S3). Such results turned out statistically significant for each scale (*p*-value < 0.001 in all cases) by performing the Seemingly Unrelated Regression equation model [102], in its modification to allow for unbalanced data proposed in [5] through the Stata “suregub” command. Eighty-seven participants were excluded from the final analysis due to missing values among the predictors, leading to a total of 965 individuals.

Socio-demographic characteristics and chronic medical conditions of the study sample are shown in Table 1.

**Table 1:** Sociodemographic characteristics of the included sample (*n* = 1052)

	Mean included (SD)
**Age in years**	50.99 (15.92)
	**n/N(%) included**
**Female gender**	810/1039 (77.96%)
**University degree**	640/1050 (60.95%)
**Country where participant is living**	
**Italy**	181/1048 (17.27%)
**Australia**	159/1048 (15.17%)
**Germany**	136/1048 (12.98%)
**France**	108/1048 (10.31%)
**South Africa**	92/1048 (8.78%)
**Netherlands**	73/1048 (6.97%)
**Sweden**	71/1048 (6.77%)
**Spain**	46/1048 (4.39%)
**Turkey**	45/1048 (4.29%)
**Indonesia**	36/1048 (3.44%)
**Macau SAR**	30/1048 (2.86%)
**Switzerland**	20/1048 (1.91%)
**United Kingdom**	12/1048 (1.15%)
**Other**	39/1048 (3.72%)
**Working before and during the pandemic**	648/1047 (61.89%)
**Lost/stopped job during the pandemic**	111/1047 (10.60%)
**Not working before the pandemic**	288/1047 (27.51%)
**Chronic medical conditions**	
**Heart disease/Hypertension**	509/1052 (48.38%)
**Pulmonary disease/ Asthma**	302/1052 (28.71%)
**Immunological disorders**	154/1052 (14.64%)
**Diabetes**	118/1052 (11.22%)
**Cancer**	46/1052 (4.37%)
**Autoimmune/ rheumatic / osteoarticular diseases**	57/1052 (5.42%)
**Gastrointestinal diseases**	26/1052 (2.47%)
**Endocrine diseases**	55/1052 (5.23%)
**Other chronic disease**	78/1052 (7.41%)
**Multiple chronic conditions**	299/1052 (28.42%)

Table S1 in the Additional file 1 shows the clinical scale values of participants with medical conditions at the four waves. Lower scores at wave 2 were observed across all items. Frequency of each chronic condition in the whole sample is shown in Additional file 1: Table S2.

Table 2 shows the means and standard errors (Standard Error of the Mean, S.E.M.) of each score in the three classes at each time-point.

**Table 2:** Mean and S.E.M. of resilience indicators in each latent class^a^

	Vulnerable	Intermediate	Resilient	Sustained-resilience class
	Mean (S.E.M.)	Mean (S.E.M.)	Mean (S.E.M.)	Mean (S.E.M.)
**PHQ Total score**	20.228 (0.563)	12.228 (0.413)	6.235 (0.243)	2.661 (0.151)
**GAD Total score**	17.347 (0.382)	10.040 (0.393)	4.874 (0.182)	1.810 (0.135)
**PCL Total score**	10.514 (0.295)	6.646 (0.253)	3.154 (0.158)	1.199 (0.073)

^a^*S.E.M.* Standard Error of the Mean. Means are constrained to be equal for each class across timepoints

The estimated probability of being sustained-resilient was 34.15%. In wave 2 a lower percentage of vulnerable and a higher percentage of resilient was shown than in the other time-points (Table 3).

**Table 3:** Estimated percentage of movers in each class for each wave

Wave	Vulnerable	Intermediate	Resilient
**1**	11.39%	22.74%	65.87%
**2**	9.38%	20.10%	70.52%
**3**	11.08%	23.76%	65.16%
**4**	12.13%	21.40%	66.47%

Significant predictors of being sustained-resilient were: older age (OR 1.040, *p*-value < 0.001), still having a job during the pandemic (OR 1.887, *p*-value 0.016), approval of the COVID-19 restrictions (OR 2.160, p-value 0.016) and a higher OSSS score (OR 1.319, p-value < 0.001). On the contrary, associated with lower odds of being in the sustained-resilient group were: female gender (OR 0.356, p-value < 0.001), job loss during pandemic (OR 0.319, p-value 0.017), having had previous mental disorders (OR 0.153, p-value < 0.001), feeling lonely (OR 0.173, p-value < 0.001), a higher fear of contamination scores (Padua Inventory) (OR 0.895, p-value < 0.001), a higher comparative value on the hedonic dimension (OR 0.673, p-value 0.001), knowing someone who had been infected by COVID-19 (OR 0.621, p-value 0.036) and whether the job of a close person was at risk of Sars-Cov-2 exposure (0.620, p-value 0.048). Logistic regression results are shown in Table 4.

**Table 4:** Results of multiple logistic regression to predict sustained resilience among included participants ^a^

	Odds ratio	Confidence interval	Standard Error
**Female gender**	**0.356**	**(0.214; 0.593)**	0.093
**Age in years**	**1.040**	**(1.021; 1.058)**	0.010
**University degree**	1.050	(0.666; 1.656)	0.244
**Square meters per person (house)**	0.995	(0.988; 1.002)	0.003
**Reference group: No job before the pandemic**			
**Working before and after pandemic start**	**1.887**	**(1.130; 3.145)**	0.495
**Losing job during pandemic**	**0.319**	**(0.125; 0.816)**	0.153
**Income reduction**	0.943	(0.572; 1.555)	0.241
**Previous mental disorder**	**0.153**	**(0.092; 0.255)**	0.040
**Approving COVID-19 restrictions**	**2.160**	**(1.156; 4.032)**	0.690
**Adhering to COVID-19 restrictions**	1.178	(0.745; 1.862)	0.275
**Going outdoor**	1.266	(0.792; 2.020)	0.303
**Knowing infected people**	**0.621**	**(0.398; 0.969)**	0.141
**COVID-19 personal job exposure**	0.747	(0.424; 1.318)	0.216
**Close person with COVID-19 job exposure**	**0.620**	**(0.387; 0.996)**	0.150
**Difficulties in meeting basic needs**	0.846	(0.512; 1.399)	0.217
**Close person with difficulties meeting basic needs**	1.656	(0.931; 2.941)	0.485
**Willingness to get vaccinated**	0.690	(0.413; 1.151)	0.180
**Baseline Padua Inventory score**	**0.895**	**(0.866; 0.926)**	0.015
**Feeling lonely**	**0.173**	**(0.088; 0.341)**	0.060
**Baseline OSSS-3 score**	**1.319**	**(1.183; 1.473)**	0.073
**PVQ1 centered (Self-direction)**	0.981	(0.740; 1.302)	0.142
**PVQ3 centered (Security)**	0.897	(0.674; 1.193)	0.130
**PVQ4 centered (Hedonism)**	**0.673**	**(0.527; 0.858)**	0.084
**PVQ5–6 centered (Benevolence)**	1.016	(0.750; 1.377)	0.157
**PVQ7 centered (Achievement)**	1.238	(0.968; 1.580)	0.155
**PVQ8 centered (Simulation)**	1.047	(0.803; 1.366)	0.143
**PVQ9 centered (Conformity)**	1.072	(0.831; 1.383)	0.139
**PVQ11 centered (Tradition)**	0.915	(0.730; 1.145)	0.105
**Conspiracy beliefs**	0.830	(0.627; 1.099)	0.119
**Pulmonary disease/Asthma**	1.054	(0.606; 1.832)	0.298
**Diabetes**	1.600	(0.836; 3.058)	0.530
**Immunological disorders**	0.932	(0.497; 1.748)	0.299
**Cancer**	0.719	(0.260; 1.988)	0.373
**Heart disease/Hypertension**	0.896	(0.518; 1.553)	0.251
**Other chronic medical condition**	0.916	(0.540; 1.555)	0.247

^a^Statistically significant predictors marked in bold

## Discussion

In this 4-wave 1-year longitudinal cross-country online survey on the mental health impact of the COVID-19 pandemic in subjects with chronic medical conditions, we found a latent class of sustained-resilience in around one third of the sample. To the best of our knowledge, this is the first study to report on frequency and predictors of long-term stress resilience to COVID-19 pandemic in the medically ill.

As expected, individuals reporting at least one chronic medical condition showed higher anxiety, depressive and post-traumatic baseline scores as compared to those without a reported illness (Additional file 1: Table S3). This is in line with the stress literature suggesting that individuals with medical conditions have a higher stress-related vulnerability [33, 82, 91] and with recent findings during COVID-19 pandemic, showing higher levels of depression and anxiety in individuals with medical conditions or a poor self-rated health status [58, 94, 95].

Sustained-resilient individuals, in spite of specific health-related stressors during the pandemic [18, 64, 88], showed a stable pattern of psychological adaptation over time. The rest of the sample followed different trajectories including, at least in one wave, scores indicating an “intermediate” or “vulnerable” condition. A similar study of 302 chronically ill people in the US found that 49% of the sample showed no or minimal depression and anxiety scores (compared to 34% in our sample) one and 3 months after the COVID-19 outbreak [20]. Although this study similarly used the PHQ-9 and the GAD-7, PCL-5 was not administered. Moreover, the proportion of sustained resilient was calculated using an operational definition (no or minimal depression or anxiety symptoms according to validated cut-offs) while our model could identify the latent sustained-resilience class, through the Mover-Stayer model (see Statistical Analysis). Lastly, as a matter of course, the shorter (3-month vs 12-month) follow-up time of the US study missed out on people showing late-onset stress.

Logistic regression showed various general and COVID-19 specific predictors of being in the sustained resilient class. Different chronic conditions did not lead to significantly different odds of belonging to the sustained-resilience group. Older male individuals had higher odds of being sustained resilient. Although older adults, in general, tend to experience greater distress after acute stressful life events, they show higher long-term resilience as compared to younger adults [7, 51]. A greater life experience, more effective coping skills and increased emotional regulation are possible explanations for a stable long-term adjustment. Widespread awareness of the increased severity and fatality of SARS-COV-2 infection in the elderly does not seem to have increased COVID-19-related stress during the pandemic in these individuals. Similarly, a recent review of 20 cross-sectional studies on the early psychological response to COVID-19 lockdowns and isolation measures by age showed a consistent lower impact in older participants [83]. Our study confirms the protective effect of age over time, even among participants with chronic medical conditions who had other reasons, besides age, to fear severe or life-threatening COVID-19 infections. A COVID-19 pandemic-specific explanation for the protective effect of age may lie in differences in needs for group social interactions (e.g., schools, clubs, gymnasiums) which have been inaccessible owing to restriction measures. Disrupted routines, academic stress, increased risk of domestic violence, and reduced access to physical and psychosocial support have, especially in youth with chronic health conditions, been cited as specific risk factors of poor resilience [79]. In line with existing literature, male gender was a predictor of being sustained-resilient. This effect of gender on long-term resilience is small but consistent across studies [7].

Being employed before and during the pandemic was found to be an important predictor of belonging to the sustained-resilient class. There is pre-pandemic evidence of a long-term protective effect of employment on mental health directly due to independence, the socially recognized job role, and economic security [31]. In contrast, people who lost their jobs during the pandemic were found to be at risk of not being sustained resilient. This figure is expected, in light of the severe economic crisis triggered by the pandemic, in people who were already concerned about their health. Unemployment and financial problems are clearly associated with poor mental health and lower quality of life [68] and recent studies have shown that individuals who lost their jobs during the COVID-19 pandemic are at risk of adverse mental health outcomes [21, 72].

A history of mental disorders was a strong predictor of not-resilient classes in our sample. During the COVID-19 pandemic, individuals with pre-existing mental disorders have been increasingly recognized as being at higher risk for adverse mental health outcomes [4, 25]. Similarly, a self-reported previous psychiatric disorder was found to be strongly associated with higher severity of COVID-19 pandemic stress symptoms [29].

With regard to the psychological variables assessed, perceived social support predicted sustained resilience while loneliness was significantly associated with non-resilient trajectories. These findings are in congruence with a large body of literature [44]. Stress and support interact and, according to the ‘stress buffering hypothesis’, the belief that support is available, reduces the impact of stressors [44]. Social isolation and loneliness increase the negative appraisal of threats, affecting stress-related vulnerability and health outcomes [16, 69]. Compared to other types of adversity, the social distance and isolation imposed during lockdowns likely made the buffering function of interpersonal contacts crucial in stress responses. On the other hand, loneliness has probably amplified the difficulties imposed by the restrictive measures of the pandemic and the fear of COVID-19 infection, especially in people with chronic medical conditions. Other studies conducted during the COVID-19 pandemic showed strong relations between lack of social support, loneliness and resilience to the psychological impact of lockdowns and isolation measures [45, 86].

Among the ten basic value orientations assessed, participants with stronger focus on hedonism values, showed a lower chance to be in the sustained-resilient group. According to the theory underlying the PVQ-10, values are defined as “desirable, trans-situational goals, varying in importance, that serve as guiding principles in people’s lives” [78]. Hedonists are those who attribute more than others a central role in pleasant and satisfying time and gratification. Hedonism belongs to a “modern” (versus “traditional”), open-to-change area of values which also includes self-direction, stimulation, achievement, and power. It has been shown that modern values are protective against stress via social sharing processes [53]. Therefore, modern values may predict resilient outcomes. For example, hedonism as measured with PVQ-10 was negatively correlated with post-traumatic stress in soldiers after military deployment [103]. In Chinese, Russian, and German university students hedonism was associated with positive mental health mediated by self-rated resilience [52]. Our results in contrast show that hedonism predicts non-resilient trajectories. We believe that satisfaction- and pleasure-oriented individuals specifically suffered from restrictions which interrupted recreational and social activities during the COVID-19 pandemic. This could be especially true in individuals suffering from additional limitations to pleasurable activities imposed by their medical condition. The presence of individual characteristics, such as values, that are associated with resilience during the pandemic in a different or opposite manner to other adversities should be addressed in future studies.

Among COVID-19-related variables, contamination fear scores of the Padua Inventory decreased the probability to be sustained-resilient. In line with recent literature [87], people with a general fear of contamination were less resilient, especially in the first months of the pandemic when governments and media reported daily raw data on infections and mortality due to COVID-19 worldwide.

Knowing someone who had been infected by COVID-19 and having a close person whose job was at risk of Sars-Cov-2 exposure were associated with non-resilient trajectories. These findings could be explained by both fear of contamination and concern for the health and safety of loved ones. In line with the latter, personal exposure did not significantly affect resilience, as if fear for close ones tended to outweigh fear for one’s own health, despite higher COVID-19 fear due to the medical condition.

Approving pandemic containment restrictions was associated with belonging to the sustained-resilience group. In line, adherence to stay-at-home orders was associated with lower declines in life satisfaction in a prospective study on adolescents during the COVID-19 pandemic [54]. COVID-19 lockdown rules and social distancing of healthy people were not understood and/or accepted by the entire population. Fear of COVID-19 infection was recognized as the prominent reason for accepting restrictions [22], but it cannot account for our result because fear often lead to anxiety symptoms. Nonetheless, adherence to COVID-19 restrictions is dependent on the extent these restrictions are perceived as effective [24] and positive attitudes such as self-efficacy and promoting one’s own health could explain the endorsement of restrictions by individuals with sustained resilience. If confirmed in specific future studies, this finding could suggest implementation of pro-adherence interventions based more on information about the benefits of containment measures than on fear of infection.

There are several important study limitations that must be considered. First, the convenience sample recruited online may lack generalizability. A self-selection bias may have occurred, e.g. subjects accepting multiple psychological assessments over 1 year are prone to proactive engagement and might be more resilient than subjects not participating. As is often the case in samples recruited through social media, most individuals were highly educated and had access to the internet. In addition, the sample is characterized by a high prevalence of middle-aged women. Second, there was a high rate of participants with fewer than three waves completed. However, the comparison of sociodemographic and clinical characteristics between included and excluded (because did not fill at least three waves of the survey) participants revealed differences only in three COVID-19-related variables (Additional file 1: Table S4). Third, although reasonably accurate [56], self-reports of chronic medical condition might represent a source of information bias. In addition, the sample consists of subjects with heterogeneous diseases. Their severity, impact, treatments and possible changes during the pandemic were not assessed in this survey.

However, some strengths of the study should be taken into account: the sample size which involves thirteen countries and its longitudinal design with four waves investigating multiple phases of the COVID-19 pandemic.

## Conclusions

In the first year of the COVID-19 pandemic, around one-third of people with a chronic medical condition showed trajectories of sustained-resilience with no significant stress over time. Some predictors were in line with the literature on stress resilience during adversities. Employed older men, without previous mental disorders, had higher chances to belong to this group, being less exposed to the impact of social restrictions during the pandemic. Loneliness and lack of social support have probably amplified the effect of restrictive measures, especially in people with chronic medical conditions. Unexpectedly, hedonism, a value considered protective against stress, was found to predict non-resilience and deserve further investigation. Fear of contamination, concern for the health of loved ones, and non-approving restrictions predicted non-resilient trajectories and are new findings.

Available technology allowed to conduct an unprecedented international study on people with chronic conditions during a global crisis. We found similarities and differences from known predictors of sustained resilience and identified some new ones specific to pandemics. Our findings add knowledge about human resilience during pandemics and may be useful in the design of resilience-building interventions in people with chronic diseases.

## Supplementary Information

**Additional file 1: **Procedures for model identification. **Fig. S1.** Flowchart of sample identification. **Table S1.** Descriptive statistics of resilience indicators across the survey waves (*N* = 1052)*. **Table S2.** Frequency of each chronic medical condition in the whole sample. **Table S3.** Comparison between participants with and without chronic medical conditions at baseline*. **Table S4.** Descriptive statistics at the first wave of socio-demographic and clinical characteristics of participants with a chronic condition included vs excluded (because did not fill at least three waves of the survey). **Table S5.** Gender distribution of participants.

## Data Availability

The data that support the findings of this study are available from the COMET Consortium, but restrictions apply to the availability of these data, which were used under license for the current study, and so are not publicly available. Data are however available upon reasonable request to the Corresponding Author, Prof. Lorenzo Tarsitani (lorenzo.tarsitani@uniroma1.it), who will forward your request to the COMET Consortium for its approval.
