# Supplementary material for: Resilience of people with chronic medical conditions during the COVID-19 pandemic: a 1-year longitudinal prospective survey
Source: BMC Psychiatry. 2022 Oct 1;22:633. doi: 10.1186/s12888-022-04265-8 (PMC9525930; doi:10.1186/s12888-022-04265-8)
Supplement: Supplementary file 1 — Additional file 1: Procedures for model identification. Fig. S1. Flowchart of sample identification. Table S1. Descriptive statistics of resilience indicators across the survey waves (N = 1052)*. Table S2. Frequency of each chronic medical condition in the whole sample. Table S3. Comparison between participants with and without chronic medical conditions at baseline*. Table S4. Descriptive statistics at the first wave of socio-demographic and clinical characteristics of participants with a chronic condition included vs excluded (because did not fill at least three waves of the survey). Table S5. Gender distribution of participants. [file 12888_2022_4265_MOESM1_ESM.docx]

**Additional file 1: Figure S1. Flowchart of sample identification**

**
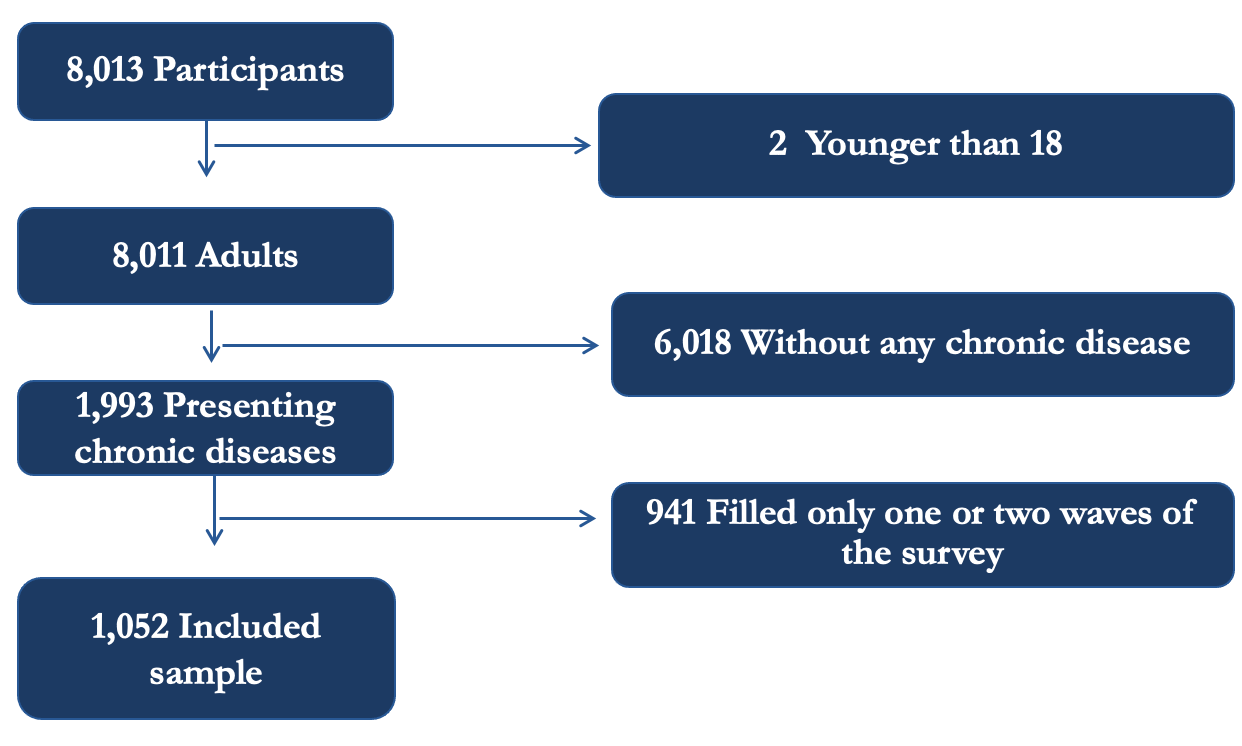
**

**Procedures for model identification**

Identification of the latent variable related to conspiracy beliefs about the Covid-19 pandemic was achieved by fixing factor variance to 1. Moreover, to avoid convergence issues, due to the high collinearity across indicators, a Rasch model (Rasch, 1980) was used (i.e, factor loadings were held equal across indicators).

In order to avoid convergence issues and to guarantee that the class ordering was the one desired (with class 1 being the one with the highest values of mean scores and class 3 the one with the lowest ones), the model was performed on a step-by-step basis, to generate starting values for the final model.

In particular, we started with a latent class model to identify resilience as a 3-class latent variable at time 1. The resulting indicator means were used as starting point for a latent transition analysis (LTA) with time 1 and 2, then the procedure was replied: results of LTA with 2 timepoints were used as starting values for the one with 3 timepoints, that were finally used for a LTA with 4 timepoints, that gave starting values for the final model. Also, the Rasch model to identify the conspiracy belief construct was performed in isolation to generate starting values both for the thresholds and for the common factor loading.

In the final model, the conspiracy beliefs indicator was regressed on the other predictors, in order to avoid the assumption of independence between them. Considering the possible computational difficulties due to variables with a variance far from the 1-10 range (Muthén & Muthén, 2017), all regression predictors with a variance above 10 or below 1 were rescaled by either multiplying or dividing them by the minimum integer value making their variance fit the (1, 10) interval, and then rescaled back to get the final estimates.

In all cases, we adopted the MPlus default numbers of initial stage random sets of starting values to generate (20) and of the number of final stage optimizations to use (4). However, to make sure that the solution found was not a local maximum, we repeated the analysis by increasing such numbers to 100 and 20 and, in case of a differing value of the best likelihood, to 200 and 40 and, in case, to 500 and 100. In all cases, the best likelihood was replicated, and results from the model with the highest numbers of starting values and of final stage optimizations were considered.

Following Mooijaart (Mooijaart, 1998) and Nylund (Nylund, 2007), stayers were set as reference class, thus the model predicted probability of not being sustained resilient, and estimates of parameters and confidence intervals were then obtained by calculating the reciprocal.

**Procedure to determine values of square meters per person**

The number of squared meters per person was calculated as the ratio between squared meters and number of people in the household. Central values of each category were considered for square meters and square feet; “larger than 200 m2” were set to 215 squared meters, while “larger than 2500 sq ft” to 2600 square feet; as for number of people in the household, “more than 5” was recoded to “6”. Square feet were converted in square meters by diving by 10.7639.

**Additional file 1: Table S1. Descriptive statistics of resilience indicators across the survey waves (N=1,052)***

|  | **Wave 1** | **Wave 2** | **Wave 3** | **Wave 4** |
| --- | --- | --- | --- | --- |
|  | **Mean (SD)** | **Mean (SD)** | **Mean (SD)** | **Mean (SD)** |
| **PHQ1** | 1.12 (0.99) | 0.92 (0.91) | 1.12 (0.95) | 1.04 (0.93) |
| **PHQ2** | 1.02 (0.96) | 0.89 (0.91) | 1.05 (0.95) | 0.98 (0.93) |
| **PHQ3** | 1.27 (1.06) | 1.12 (0.99) | 1.30 (1.04) | 1.25 (1.03) |
| **PHQ4** | 1.36 (1.02) | 1.24 (0.95) | 1.37 (0.97) | 1.36 (0.98) |
| **PHQ5** | 1.07 (1.06) | 0.85 (0.96) | 1.01 (1.03) | 0.94 (1.00) |
| **PHQ6** | 0.77 (1.02) | 0.70 (0.95) | 0.74 (0.95) | 0.75 (0.98) |
| **PHQ7** | 0.89 (0.96) | 0.77 (0.91) | 0.85 (0.96) | 0.81 (0.92) |
| **PHQ8** | 0.42 (0.77) | 0.33 (0.67) | 0.38 (0.73) | 0.36 (0.71) |
| **PHQ9** | 0.26 (0.65) | 0.24 (0.62) | 0.24 (0.61) | 0.26 (0.65) |
| **GAD1** | 1.14 (0.93) | 0.99 (0.92) | 1.12 (0.94) | 1.06 (0.92) |
| **GAD2** | 0.88 (0.96) | 0.78 (0.92) | 0.89 (0.96) | 0.85 (0.96) |
| **GAD3** | 0.99 (0.93) | 0.88 (0.92) | 0.99 (0.95) | 0.94 (0.93) |
| **GAD4** | 1.08 (0.97) | 0.98 (0.94) | 1.09 (0.96) | 1.07 (0.95) |
| **GAD5** | 0.48 (0.79) | 0.45 (0.76) | 0.50 (0.81) | 0.49 (0.79) |
| **GAD6** | 0.99 (0.92) | 0.87 (0.86) | 0.98 (0.90) | 0.92 (0.88) |
| **GAD7** | 0.79 (0.92) | 0.67 (0.89) | 0.78 (0.94) | 0.68 (0.88) |
| **PCL1** | 0.88 (1.10) | 0.82 (1.05) | 0.88 (1.09) | 0.91 (1.08) |
| **PCL2** | 0.97 (1.14) | 0.86 (1.09) | 0.95 (1.13) | 0.93 (1.11) |
| **PCL3** | 1.53 (1.26) | 1.20 (1.18) | 1.39 (1.25) | 1.41 (1.29) |
| **PCL4** | 0.91 (1.05) | 0.75 (0.94) | 0.92 (1.06) | - 1. (1.02) |

*PHQ=Patient Health Questionnaire, GAD=Generalized Anxiety Disorder scale, PCL=PTSD Checklist DSM-5. The first wave of the survey took place between May and July 2020. The second wave took place between September and October 2020, the third wave took place in December 2020, while the fourth wave took place between March and April 2021.

**Additional file 1: Table S2. Frequency of each chronic medical condition in the whole sample**

|  |  |
| --- | --- |
| Heart disease/Hypertension | 619/8,011 (7.73%) |
| Pulmonary disease/ Asthma | 382/8,011 (4.77%) |
| Immunological disorders | 157/8,011 (1.97%) |
| Diabetes | 76/8,011 (0.949%) |
| Cancer | 52/8,011 (0.649%) |
| Other | 253/8,011 (3.16%) |
| Multiple conditions | 454/8,011 (5.67%) |

**Additional file 1: Table S3. Comparison between participants with and without chronic medical conditions at baseline***

|  | **Time 1 Participants with chronic medical condition(s)** | **Time 1 Participants without any chronic medical condition(s)** | **Estimated effect of chronic condition** | **SUR p-value** |
| --- | --- | --- | --- | --- |
|  | **Mean (SD)** | **Mean (SD)** | **Coef.** | **<0.001** |
| **PHQ1** | 1.117 (0.988) | 1.057 (0.920) | .067 |  |
| **PHQ2** | 1.018 (0.958) | 0.916 (0.889) | .112 |  |
| **PHQ3** | 1.267 (1.056) | 1.082 (1.015) | .192 |  |
| **PHQ4** | 1.361 (1.016) | 1.187 (0.933) | .181 |  |
| **PHQ5** | 1.067 (1.059) | 0.827 (0.956) | .245 |  |
| **PHQ6** | 0.769 (1.024) | 0.712 (0.962) | .061 |  |
| **PHQ7** | 0.886 (0.965) | 0.779 (0.928) | .111 |  |
| **PHQ8** | 0.418 (0.769) | 0.324 (0.650) | .097 |  |
| **PHQ9** | 0.264 (0.647) | 0.198 (0.578) | .070 |  |
| **GAD1** | 1.143 (0.933) | 1.014 (0.888) | .137 | **<0.001** |
| **GAD2** | 0.879 (0.955) | 0.747 (0.898) | .145 |  |
| **GAD3** | 0.989 (0.934) | 0.865 (0.910) | .135 |  |
| **GAD4** | 1.076 (0.971) | 0.959 (0.927) | .129 |  |
| **GAD5** | 0.475 (0.785) | 0.442 (0.737) | .040 |  |
| **GAD6** | 0.994 (0.922) | 0.903 (0.850) | .098 |  |
| **GAD7** | 0.785 (0.921) | 0.615 (0.846) | .176 |  |
| **PCL1** | 0.878 (1.101) | 0.752 (0.989) | .124 | **<0.001** |
| **PCL2** | 0.970 (1.139) | 0.809 (1.066) | .158 |  |
| **PCL3** | 1.526 (1.262) | 1.318 (1.206) | .203 |  |
| **PCL4** | 0.908 (1.046) | 0.826 (1.005) | .082 |  |

*** SUR = Seemingly Unrelated Regression**

**Additional file 1: Table S4. Descriptive statistics at the first wave of socio-demographic and clinical characteristics of participants with a chronic condition included vs excluded (because did not fill at least three waves of the survey)**

|  | **Mean included (SD)** | **Mean excluded (SD)** |
| --- | --- | --- |
| **PHQ1** | 1.12 (0.99) | 1.28 (1.01) |
| **PHQ2** | 1.02 (0.96) | 1.18 (1.00) |
| **PHQ3** | 1.27 (1.06) | 1.44 (1.11) |
| **PHQ4** | 1.36 (1.02) | 1.48 (1.02) |
| **PHQ5** | 1.07 (1.06) | 1.25 (1.09) |
| **PHQ6** | 0.77 (1.02) | 0.89 (1.08) |
| **PHQ7** | 0.89 (0.96) | 0.96 (1.01) |
| **PHQ8** | 0.42 (0.77) | 0.50 (0.82) |
| **PHQ9** | 0.26 (0.65) | 0.34 (0.72) |
| **GAD1** | 1.14 (0.93) | 1.26 (0.99) |
| **GAD2** | 0.88 (0.96) | 1.04 (1.04) |
| **GAD3** | 0.99 (0.93) | 1.14 (1.03) |
| **GAD4** | 1.08 (0.97) | 1.19 (1.05) |
| **GAD5** | 0.48 (0.79) | 0.66 (0.92) |
| **GAD6** | 0.99 (0.92) | 1.12 (0.97) |
| **GAD7** | 0.79 (0.92) | 0.99 (1.04) |
| **PCL1** | 0.88 (1.10) | 1.09 (1.18) |
| **PCL2** | 0.97 (1.14) | 1.19 (1.21) |
| **PCL3** | 1.53 (1.26) | 1.72 (1.33) |
| **PCL4** | 0.91 (1.05) | 1.07 (1.14) |
| **Age in years** | 50.998 (15.920) | 49.134 (15.628) |
| **Squared meters per person (house)** | 52.339 (33.890) | 50.991 (34.122) |
| **PAD1** | 2.440 (1.268) | 2.635 (1.345) |
| **PAD2** | 1.922 (1.102) | 2.176 (1.234) |
| **PAD3** | 2.251 (1.170) | 2.461 (1.256) |
| **PAD4** | 2.183 (1.231) | 2.383 (1.305) |
| **PAD5** | 2.517 (1.358) | 2.800 (1.415) |
| **PAD6** | 2.500 (1.486) | 2.800 (1.581) |
| **PAD7** | 2.688 (1.328) | 2.840 (1.332) |
| **PAD8** | 1.889 (1.153) | 2.037 (1.215) |
| **PAD9** | 2.526 (1.312) | 2.723 (1.332) |
| **PAD10** | 1.509 (0.946) | 1.598 (0.989) |
| **PVQ1 centered** | -0.644 (1.158) | -0.699 (1.174) |
| **PVQ3 centered** | -0.398 (1.175) | -0.248 (1.159) |
| **PVQ4 centered** | 0.153 (1.136) | 0.002 (1.100) |
| **PVQ5-6 centered** | -0.940 (0.914) | -1.005 (0.876) |
| **PVQ7 centered** | 0.373 (1.131) | 0.430 (1.113) |
| **PVQ8 centered** | 0.839 (1.199) | 0.950 (1.127) |
| **PVQ9 centered** | -0.253 (1.233) | -0.355 (1.188) |
| **PVQ11 centered** | 0.469 (1.355) | 0.647 (1.329) |
| **OSS-S2** | 2.821 (0.804) | 2.788 (0.844) |
| **OSS-S3** | 3.457 (1.115) | 3.501 (1.120) |
| **OSS-S4** | 2.913 (1.151) | 2.998 (1.140) |
|  | **n/N(%) included** | **n/N(%) excluded** |
| **Female gender** | 810/1039 (77.96%) | 690/934 (73.88%) |
| **University degree** | 640/1,050 (60.95%) | 517/939 (55.06%) |
| **Working before and during the pandemic** | 648/1,047 (61.89%) | 612/940 (65.11%) |
| **Lost/stopped job during the pandemic** | 111/1,047 (10.60%) | 124/940 (13.19%) |
| **Not working before the pandemic** | 288/1,047 (27.51%) | 204/940 (21.70%) |
| **Income Reduction during the pandemic** | 303/1,041 (29.11%) | 370/930 (39.78%) |
| **Previous mental disorder** | 318/1,050 (30.29%) | 303/939 (32.27%) |
| **Approving COVID-19 restrictions** | 893/1,048 (85.21%) | 753/938 (80.28%) |
| **Adhering to COVID-19 restrictions** | 752/1,045 (71.96%) | 638/936 (68.16%) |
| **Going outdoor** | 669/1,044 (64.08%) | 527/935 (56.36%) |
| **Knowing infected people** | 510/1,047 (48.71%) | 370/937 (39.49%) |
| **Personal job exposure to COVID-19** | 263/1,052 (25.00%) | 228/941 (24.23%) |
| **Close person with COVID-19 job exposure** | 362/1,052 (34.41%) | 308/941 (32.73%) |
| **Personal difficulty meeting basic needs** | 352/1,052 (33.46%) | 317/941 (33.69%) |
| **Close person with difficulties meeting basic needs** | 160/1,052 (15.21%) | 149/941 (15.83%) |
| **Willingness to get vaccinated** | 683/1,046 (65.30%) | 142/232 (61.21%) |
| **Small group manipulating** | 392/981 (39.96%) | 140/295 (47.46%) |
| **Scientists manipulating** | 396/981 (40.37%) | 126/295 (42.71%) |
| **Laboratory weapon** | 412/981 (42.00%) | 155/295 (52.36%) |
| **Feeling lonely** | 264/1,050 (25.14%) | 239/939 (25.45%) |

As for the comparison of sociodemographic and clinical characteristics to predict resilience of the sample of repliers vs the one of non-repliers, the former showed lower mean values in all PAD items, and underwent income reduction or showed beliefs in a small group manipulating world events less often, while they went out at least 3 times per week in the 2 weeks before the first measurement or already knew someone infected more often than non-repliers

**Additional file 1: Table S5. Gender distribution of participants**

|  | **n** |
| --- | --- |
| **Female** | 689 |
| **Male** | 244 |
| **Transgender female** | 1 |
| **Transgender male** | 0 |
| **Not listed** | 2 |
| **Prefer not to answer** | 2 |
| **Total** | 938 |

References

Mooijaart, A. (1998). Log-linear and Markov modeling of categorical longitudinal data. In *Longitudinal data analysis: Designs, models, and methods* (pp. 318–370).

Muthén, L. K., & Muthén, B. O. (2017). *Mplus User’s Guide (Eight edition).* (Muthén & Muthén).

Nylund, K. L. (2007). Latent transition analysis: Modeling extensions and an application to peer victimization (Doctoral dissertation). In *University of California Los Angeles*.

Rasch, G. (1980). *Probabilistic models for some intelligence and attainment tests*. Chicago, IL: University of Chicago Press. (Original work published 1960).
